# Theoretical Prediction and Experimental Validation of the Glass-Forming Ability and Magnetic Properties of Fe-Si-B Metallic Glasses from Atomic Structures

**DOI:** 10.3390/ma15093149

**Published:** 2022-04-27

**Authors:** Yuhang Jiang, Shangke Jia, Shunwei Chen, Xuelian Li, Li Wang, Xiujun Han

**Affiliations:** 1 School of Materials Science and Engineering, Qilu University of Technology (Shandong Academy of Sciences), Jinan 250353, China; yhjiang-qlu@139.com (Y.J.); shangkejia@outlook.com (S.J.); swchen@qlu.edu.cn (S.C.); 2 School of Mechanical and Electrical Engineering, Shandong University, Weihai 264209, China; wanglihxf@sdu.edu.cn

**Keywords:** Fe-based amorphous alloys, ab initio molecular dynamics simulations, glass-forming ability, soft magnetic property

## Abstract

Developing new soft magnetic amorphous alloys with a low cost and high saturation magnetization (*B*_s_) in a simple alloy system has attracted substantial attention for industrialization and commercialization. Herein, the glass-forming ability (GFA), thermodynamic properties, soft magnetic properties, and atomic structures of Fe_80+x_Si_5−x_B_15_ (*x* = 0–4) amorphous soft magnetic alloys were investigated by ab initio molecular dynamics (AIMD) simulations and experiments. The pair distribution function (PDF), Voronoi polyhedron (VP), coordination number (CN), and chemical short- range order (CSRO) were analyzed based on the AIMD simulations for elucidating the correlations between the atomic structures with the glass-forming ability and magnetic properties. For the studied compositions, the Fe_82_Si_3_B_15_ amorphous alloy was found to exhibit the strongest solute–solute avoidance effect, the longest Fe-Fe bond, a relatively high partial CN for the Fe-Fe pair, and the most pronounced tendency to form more stable clusters. The simulation results indicated that Fe_82_Si_3_B_15_ was the optimum composition balancing the saturation magnetization and the GFA. This prediction was confirmed by experimental observations. The presented work provides a reference for synthesizing new Fe-Si-B magnetic amorphous alloys.

## 1. Introduction

Fe-based amorphous alloys have excellent soft magnetic properties, high saturation magnetization (*B*_s_), and low coercivity (*H*_c_) [1,2,3,4,5,6]. In addition, they have good mechanical properties and a low price. Therefore, Fe-based amorphous alloys have important potential applications in the field of energy conversion, especially in the power industry [7,8,9]. To reduce energy consumption and the price, many types of amorphous distribution transformers have been developed and launched into the market. Fe-based amorphous alloys have been applied to replace traditional Si-steel and ferrite in fabricating the core.

However, there are still two important challenges to the industrial application of Fe-based soft magnetic amorphous alloys [10]. Firstly, the glass-forming ability (GFA) of Fe-based alloys is unsatisfying. At present, an amorphous alloy transformer core is made by winding and stacking amorphous ribbons, which leads to eddy currents in the joints. Therefore, the improvement of the GFA is important to fabricate thicker amorphous ribbons in order to improve the energy conversion efficiency by reducing the stacking number of the amorphous strips. Secondly, the *B*_s_ value of Fe-based soft magnetic alloys needs to be improved. However, the *B*_s_ and GFA are normally in contradiction with each other; that is, increasing the Fe content can increase the *B*_s_, but the GFA will deteriorate at the same time. It is rather difficult to concurrently obtain an alloy with the highest saturation magnetization and the best GFA. Therefore, it is important to find a composition that can balance the *B*_s_ and GFA. The optimum composition should have the highest *B*_s_ and a relatively good GFA.

In the past several decades, many attempts have been made to improve the *B*_s_ and many types of Fe-based amorphous alloys have been developed [11,12,13,14], especially Co- and P-containing Fe-based alloys, whose *B*_s_ could reach 1.92 T [14]. However, the existence of Co and P prevents their application because of the expensive cost of the raw materials and/or the processing of the pre-alloy ingots. Therefore, it is important to develop soft magnetic amorphous alloys with a high *B*_s_ in simple Fe-based alloy systems without Co and P elements. Until now, the most popular materials in the power industry have been Fe-Si-B series such as Fe_78–80_Si_9–11_B_11–13_ (Metglas2605SA1) [7] and Fe_82_Si_4_B_13_C_1_ (Metglas2605HB1) [15], which only have a *B*_s_ of 1.57 and 1.64 T, respectively. The further improvement of *B*_s_ of Fe-Si-B alloys is, therefore, still meaningful and open to investigation.

According to previous studies, the atomic structure has important influences on the GFA and magnetic properties [16,17,18,19,20,21]. The atomic structure is mainly characterized by two types of short-range ordering in the first nearest coordination spheres; namely, the topological short-range order (TSRO) and the chemical short-range order (CSRO). The TSRO indicates a tendency to form a certain coordination of atoms in space whereas the CSRO characterizes the tendency of an atom to surround itself with the same or different atoms. Several studies have shown that the solute–solute avoidance effect—namely, solute atoms not situated around the centered solvent atom—could stabilize the cluster and, therefore, improve the GFA [16,17,18]. The solute–solute avoidance effect is a type of CSRO that also plays an important role in affecting the magnetic properties. Vincze et al. found that, in an Fe-B amorphous alloy, the magnetic moment of the Fe atom correlated with the number of metalloid atoms in the first nearest neighbor shell [19].

The question of whether the optimum composition of an Fe-based amorphous alloy can be reasonably predicted from its atomic local structure, especially the CSRO, is of great interest. To address this question, in this work ab initio molecular dynamics (AIMD) simulations were carried out using the Fe-Si-B ternary system as a model. The correlations between the atomic structure with the GFA and magnetic properties were explored. To verify the validity of the simulation results, Fe_80+x_Si_5−x_B_15_ (*x* = 0–4) amorphous ribbons were prepared by a single roller melt-spinning method. The GFA, thermal stability, and soft magnetic properties were studied by X-ray powder diffraction (XRD), differential scanning calorimetry (DSC), and vibrating sample magnetometer (VSM), respectively. Based on the simulation and experimental results, the Fe_82_Si_3_B_15_ amorphous alloy was determined to be the optimum composition with a relatively good GFA and thermal stability as well as the best soft magnetic properties amongst our developed samples. The presented results in this work provide a reference for synthesizing new Fe-Si-B amorphous alloys with a superior soft magnetic performance and a good GFA.

## 2. Simulation and Experimental Details

AIMD simulations were used to gain an insight into the local atomic structure, employing a generalized gradient approximation (GGA) [22,23] with a Perdew–Burke–Ernzerhof (PBE) formalism based on the density functional theory (DFT) as implemented in the Vienna Ab Initio Simulation Package (VASP 5.4.1) [24,25,26,27]. The simulated configuration of the Fe_80+x_Si_5−x_B_15_(*x* = 0–4) amorphous alloys contained 100 atoms in a cubic cell with periodic boundary conditions. The simulation was performed in a canonical ensemble with a Nosé thermostat. The equation of motion was solved via a velocity Verlet algorithm with a timestep of 4 fs. Only the Γ point was applied to the sampling in the Brillion zone of the supercell. The systems were firstly melted at T = 2100 K for 4 ps and then quenched to T = 300 K with a cooling rate of 4 × 10^13^ K/s. The volume of the system was adjusted to correspond with zero total pressure. The last 5000 configurations were collected for the statistics.

To verify the simulation results, alloy ingots with a nominal composition of Fe_80+x_Si_5−x_B_15_ (*x* = 0–4) were prepared by induction melting into the mixtures of Fe (99.99 wt%), the commercial Fe-B master alloy (19.35 wt% B), and Si (99.99 wt%) in a highly purified argon atmosphere. Before the induction melting, the vacuum chamber was evacuated to 1 × 10^−3^ Pa and then backfilled with high-purity argon. This evacuation–washing process was repeated three times to ensure the removal of the residual oxygen. To ensure compositional homogeneity, the alloy ingots were melted 4–5 times. The melt-spun ribbons, with a thickness of 20–30 μm and width of 1 mm, were prepared by a single roller melt-spinning method under a high-purity argon atmosphere.

The structures of the melt-spun ribbons were identified by XRD with Cu Kα radiation. The thermal stability parameters of the amorphous alloys, including the onset crystallization temperature (*T*_x_), melting temperature (*T*_m_), and liquidus temperature (*T*_l_), were examined by DSC at a heating rate of 0.67 K/s. The *B*_s_ value was measured by VSM under an applied field of 1600 kA/m at room temperature. The *H*_c_ was measured under a field of 800 A/m with a DC B-H loop tracer. The density (*ρ*) was measured by the Archimedean method using pure water as the fluid.

## 3. Results and Discussion

### 3.1. AIMD Simulations

AIMD simulations have been widely used to study the atomic structure of metallic glasses [28,29]. The TSRO and CSRO of the Fe_80+x_Si_5−x_B_15_ (*x* = 0–4) metallic glasses were investigated by AIMD simulations and the correlations between their GFA and magnetic properties were explored.

**Figure 1:**
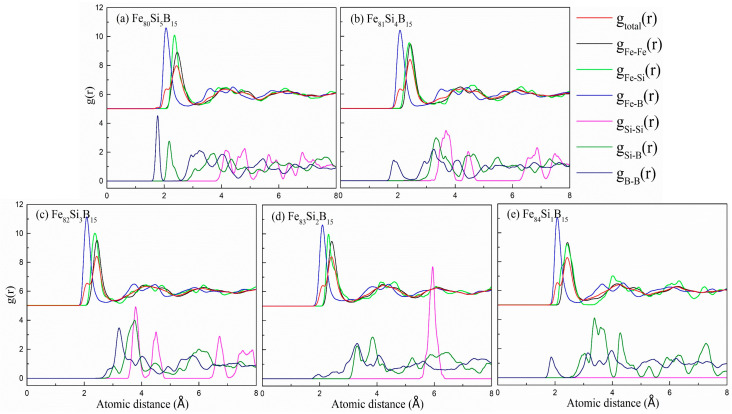
Total and partial PDFs of the Fe_80+x_Si_5−x_B_15_ (*x* = 0–4) amorphous alloys at 300 K. (**a**) Fe_80_Si_5_B_15_, (**b**) Fe_81_Si_4_B_15_, (**c**) Fe_82_Si_3_B_15_, (**d**) Fe_83_Si_2_B_15_ and (**e**) Fe_84_Si_1_B_15_.

The total and partial pair distribution functions (PDFs) of the Fe_80+x_Si_5−x_B_15_ (*x* = 0–4) amorphous alloys at 300 K are plotted in Figure 1, in which all characteristic broad peaks can be observed, demonstrating the appearance of an amorphous structure. The positions of the first peaks of *g*_total_(r) for the different compositions showed mere changes at 2.4 Å. The first peaks of *g*_Fe-M_(r) were intense, indicating the strong interactions between the Fe and metalloid (M = Si, B) atoms. Noticeably, there existed a shoulder peak at around 2.1 Å in the first peak of *g*_total_(r) for all the compositions. Interestingly, the first peak of *g*_Fe-B_(r) was also located at the same position, indicating that the shoulder peak resulted from a relatively large amount of Fe-B neighboring pairs with a considerably shorter distance than the Fe-Fe and Fe-Si pairs. These observations were consistent with the results in the literature for Fe_78_Si_9_B_13_ [30], Fe_82_Si_4_B_10_P_4_ [24], and Fe_85_Si_2_B_9_P_4_ [31]. For all the compositions, the first peak of *g*_Fe-B_(r) for the Fe_82_Si_3_B_15_ amorphous alloy was the highest, indicating that the Fe–B interaction was the strongest in Fe_82_Si_3_B_15_ due to the strong chemical bond. The *g*_Si-Si_(r), *g*_Si-B_(r), and *g*_B-B_(r) curves possessed negligible first peaks, suggesting the presence of solute–solute avoidance [16,32,33]. The full solute–solute avoidance stabilized the alloy system in both the melted and amorphous states by forming relatively stable atomic clusters, which decreased the constituent segregation and increased the GFA [18]. As shown in Figure 1, there were almost no first peaks in the *g*_Si-Si_(r), *g*_Si-B_(r), and *g*_B-B_(r) curves for the Fe_82_Si_3_B_15_ alloy, indicating its better solute–solute avoidance compared with the other compositions; this indicated that it had a good thermal stability and GFA.

**Figure 2:**
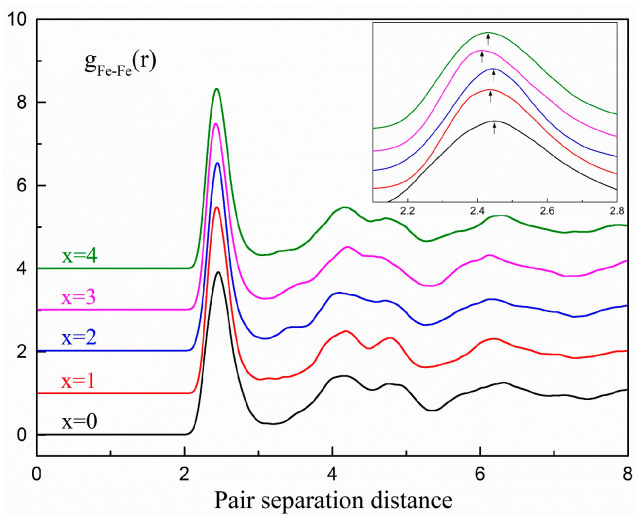
Partial PDFs for Fe-Fe pairs in the Fe_80+x_Si_5−x_B_15_ (*x* = 0–4) amorphous alloys at 300 K. The insert is the enlarged part of the first peaks.

The partial Fe-Fe PDFs for the five compositions at 300 K are shown in Figure 2 and the positions of the first peaks for *g*_Fe-Fe_(r) and *r*_Fe-Fe_ are listed in Table 1. Fe_82_Si_3_B_15_ and Fe_80_Si_5_B_15_ had an equivalent *r*_Fe-Fe_, the largest in all compositions, indicating that the Fe-Fe bond for these two compositions was the longest. The longer Fe-Fe bond favored the larger size of the Fe-centered cluster, in which more Fe and metalloid atoms could be contained. Considering that clusters with a larger size are unfavorable for the diffusion of atoms, we predicted that the GFA of Fe_82_Si_3_B_15_ should be high. In addition, the long bond between Fe atoms is beneficial to the magnetic properties [20,34]; therefore, we speculated that the Fe_80_Si_5_B_15_ and Fe_82_Si_3_B_15_ alloys might also have higher magnetic properties than the other three compositions.

To further study the topological short-range order of the structure, a Voronoi polyhedron (VP) analysis was carried out [35,36]. The position of the first minimum (at ~3.1 Å) after the first peak in the g_total_(r) curve was taken as the cut-off range for the atomic neighboring. The VP index was defined as (*n*_3_, *n*_4_, *n*_5_, and *n*_6_), in which *n*_i_ denoted the number of *i*-sided faces of the VP.

**Figure 3:**
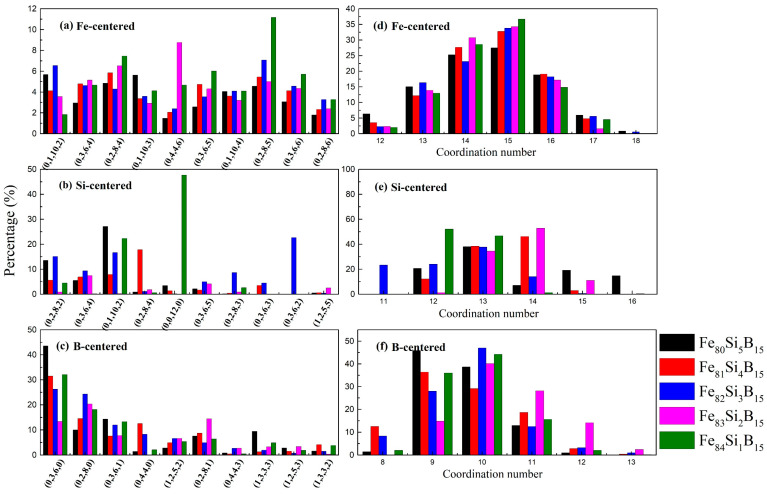
Distributions of typical VP indices for (**a**) Fe-, (**b**) Si-, and (**c**) B-centered clusters and the corresponding distributions of the CNs for (**d**) Fe-, (e) Si-, and (**f**) B-centered in the Fe_80+x_Si_5−x_B_15_ (*x* = 0–4) alloys at 300 K. The VP is defined as the polyhedron with minimum volume constituted by the vertical bisected surfaces between neighboring atoms.

The major types of VP indices with Fe- and M-centered clusters and the corresponding distribution of coordination numbers (CNs) of the Fe_80+x_Si_5__−x_B_15_ (*x* = 0–4) alloys are shown in Figure 3. As shown in Figure 3a, the (0, 1, 10, 2), (0, 2, 8, 4), and (0, 3, 6, 4) polyhedrons had the maximum proportion. Derived polyhedrons such as (0,1,10, 3) and (0, 1, 10, 4), which occupied similar rates, were also found around the Fe atoms. The Si-centered (0, 1, 10, 2) and (0, 3, 6, 4) clusters accounted for a large proportion, similar to the cluster types of the Fe-centered ones, as shown in Figure 3b. In the amorphous Fe_84_Si_1_B_15_ alloy, amongst the Si-centered VP, the (0, 0, 12, 0) type had the maximum ratio of 47.6%, probably due to the single Si atom, resulting in the non-uniformity of its structure in the Fe_84_Si_1_B_15_ alloy. In Figure 3c, amongst the B-centered VP, the (0, 3, 6, 0), (0, 2, 8, 0), and (0, 3, 6, 1) polyhedrons played a major role. Most of these conformed with the behavior of Fe_78_Si_9_B_13_ [32] and Fe_82_Si_4_B_10_P_4_ [24] metallic glasses.

The CN was calculated based on the basic theory of a VP partition. The number of facets on the VP surface (practically *n*_3_ + *n*_4_ + *n*_5_ + *n*_6_) was equal to the number of the neighboring atoms—i.e., the CN—because each facet represented the boundary of a neighboring atom. The distributions of CNs in the Fe-, Si-, and B-centered clusters of the Fe_80+x_Si_5−x_B_15_ (*x* = 0–4) amorphous alloys are illustrated in Figure 3. The Fe-centered clusters showed dominant CNs of 14 and 15, mainly attributed to the (0, 2, 8, 4), (0, 2, 8, 5), (0, 3, 6, 5), and (0, 3, 6, 6) polyhedrons. As can be seen in Figure 3e, the Si-centered cluster had a major CN of 13, which was mainly due to the (0, 1, 10, 2) polyhedron. The CN of the B-centered ones was 10 because of the (0, 2, 8, 0) polyhedron. According to the spatial connectivities between the clusters, the large clusters such as (0, 2, 8, 4) and (0, 1, 10, 2) had a strong tendency to connect to the small clusters (e.g., (0, 2, 8, 0), (0, 3, 6, 0), and (0, 4, 4, 0)), leading to dense cluster packing [37]. It could be concluded that there was a strong connection tendency between the larger clusters (Fe-centered) and the smaller ones (Si- and B-centered).

To further analyze the influence of the local structure on the GFA and atomic magnetic moment, the total and partial CNs for the Fe_80+x_Si_5−x_B_15_ (*x* = 0–4) alloys were counted; the results are presented in Table 2, Table 3 and Table 4. Evidently, in the five compositions, the partial CNs of Si-Si, Si-B, B-Si, and B-B were the least in the Fe_82_Si_3_B_15_ alloy. This indicated that Fe_82_Si_3_B_15_ had the strongest solute–solute avoidance and thus had the best GFA, which was consistent with the findings from the PDFs. In addition, according to Heisenberg [38,39], the atomic magnetic moment depends on the number of Fe atoms in the nearest neighbor (NN) shell; that is, more nearest neighbor Fe atoms correspond with higher atomic magnetic moments. As shown in Table 2, the partial CN of the Fe-Fe pair in Fe_84_Si_1_B_15_ and Fe_82_Si_3_B_15_ were large (12.8 and 12.5) and that of Fe_80_Si_5_B_15_ was the smallest (12.1). From this point of view, the Fe_84_Si_1_B_15_ and Fe_82_Si_3_B_15_ alloys should have had higher atomic magnetic moments than the other compositions. Considering the above-mentioned length of the Fe-Fe bond, the Fe_82_Si_3_B_15_ alloy was speculated to have the highest *B*_s_.

**Figure 4:**
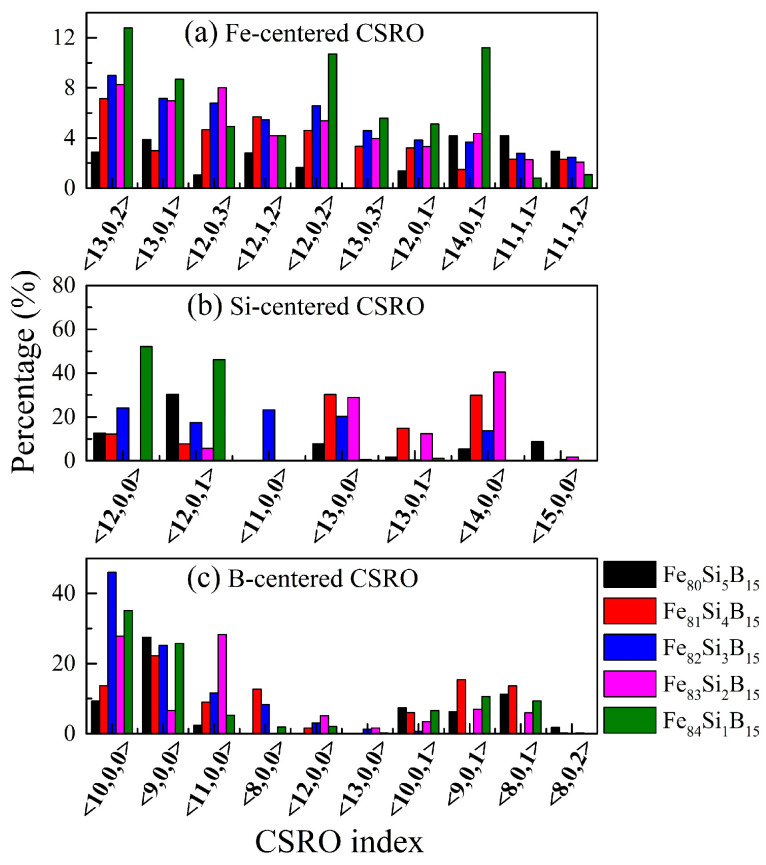
Fractions of the Fe- and M-centered CSROs in the Fe_80+x_Si_5−x_B_15_ (*x* = 0–4) alloys at 300 K: (**a**) Fe-centered; (**b**) Si-centered; and (**c**) B-centered.

The fraction of atom-centered CSROs, confined to the NN shell, is shown in Figure 4 and Table 5. The index <*n*_1_, *n*_2_, *n*_3_, *n*_4_> along the lateral axis defined the CSRO type, in which *n_i_* indicated the total number of Fe, Si, or B atoms in the chemical component. For instance, index <13, 0, 2> in Figure 4a denotes that this type of Fe-centered CSRO in the Fe_80+x_Si_5−x_B_15_ (*x* = 0–4) amorphous alloys contained 13 Fe atoms, 0 Si atom, and 2 B atoms. In the M-centered CSROs, when the same M atom existed in the NN shell, the CSRO was defined as the S-type. If the atoms in the NN shell were all Fe atoms, then this CSRO was called a P-type CSRO [18].

From Figure 4 and Table 5, it could be seen that, although the same M atoms were found in the NN shell of M, most of the surrounding atoms were Fe, indicating the formation of M-centered clusters in the Fe_80+x_Si_5−x_B_15_ (*x* = 0–4) alloys. In addition, by decreasing the Si content to 3 at.%, the fractions of the S-type reached a minimum of 0.0 and 0.6% for the Si-centered and B-centered CSROs, respectively. This indicated that the same atoms were rarely found around the Si or B atoms, resulting in an increased possibility of surrounding by the Fe atoms, which implied that the Si and B atoms had a dispersed distribution and that the solute–solute avoidance effect for the Fe_82_Si_3_B_15_ amorphous alloy was the strongest. On the other hand, Fe-centered clusters that were only surrounded by Fe atoms (P-type) were rarely found, which showed the presence of few pure Fe clusters. Thus, the Fe atoms were mainly located in the NN atoms of all elements, making the distribution of Fe atoms more uniform. With the increase in Fe content (80 at.% → 84 at.%), the proportion of Fe-centered P-type CSROs increased from 2.5 to 8.9%, which indicated that the content of pure Fe clusters increased with the Fe content.

In connection with the above-mentioned results of the PDF, VP, CN, and CSRO, we concluded that, in the Fe_80+x_Si_5−x_B_15_ (*x* = 0–4) amorphous alloys, the Fe_82_Si_3_B_15_ alloy was the optimum composition, combining a good thermal stability with the GFA and magnetic properties. To further verify the simulation results, the Fe-based amorphous alloys were prepared by specific experiments, and XRD, DSC, and VSM analyses were used for the determination of the structure, thermophysical properties, and magnetism, respectively.

### 3.2. Experimental Determination

**Figure 5:**
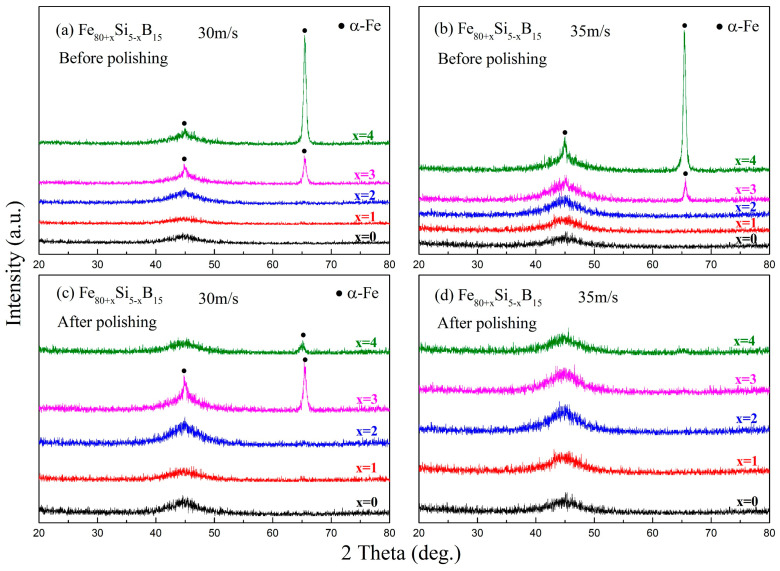
XRD patterns for the free surface of the Fe_80+x_Si_5__−x_B_15_ (*x* = 0–4) melt-spun ribbons (**a**,**b**) before and (**c**,**d**) after polishing the surface layer. The alloys are prepared with a linear velocity of 30 or 35 m/s.

The melt-spun ribbons of the Fe_80+x_Si_5__−x_B_15_ (*x* = 0–4) alloys were prepared by single roller melt-spinning with a linear velocity of 30 or 35 m/s under a highly purified argon atmosphere. XRD was used to analyze the phase structure of these ribbons (Figure 5). Before polishing, an amorphous structure was formed in the ribbons with an Fe content below 82 at.%, but further increasing the Fe content induced the formation of the α-Fe phase, as shown in Figure 5a,b. After polishing, the α-Fe phase could still be found in the ribbons with a high Fe content that were prepared at the linear velocity of 30 m/s, but only one amorphous broad peak could be found in all ribbons prepared at the linear velocity of 35 m/s, as illustrated in Figure 5c,d. The existence of the α-Fe phase was due to the crystallization layer on the free surface of the melt-spun ribbons, which was an indication of a decreased GFA. It should be noted that, when further increasing the Fe content, the GFA of the alloys was worse, as reported in the literature [40,41]. Thus, according to the XRD results, we concluded that the formation of the amorphous phase was affected by the Fe content and that the GFA decreased noticeably if the Fe content exceeded 82 at.%. For Fe_80_Si_5_B_15_, Fe_81_Si_4_B_15_, and Fe_82_Si_3_B_15_, their GFA could not be distinguished based on the XRD results. However, it was reasonable to suppose that Fe_82_Si_3_B_15_ had a relatively good GFA in the five compositions covered in this work.

**Figure 6:**
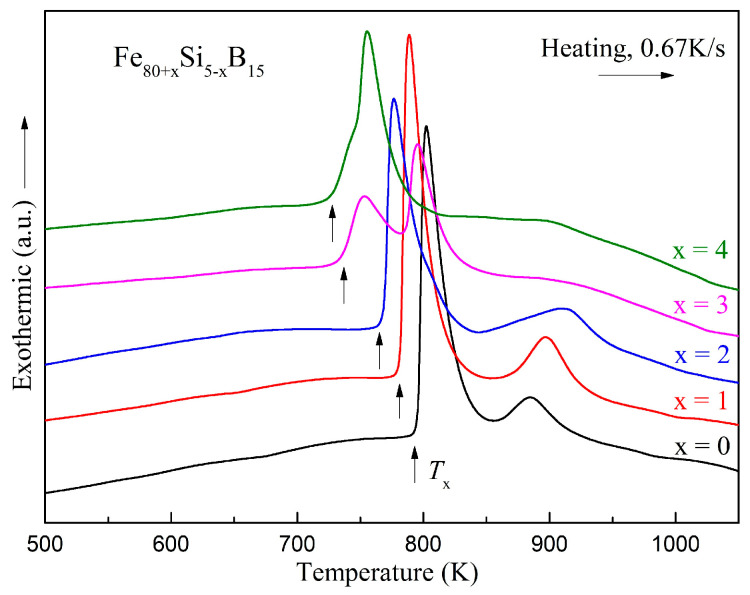
DSC curves of the Fe_80+x_Si_5−x_B_15_ (x = 0–4) amorphous ribbons.

To quantitatively characterize the thermodynamic properties of the ribbons, the polished Fe_80+x_Si_5−x_B_15_ (*x* = 0–4) amorphous ribbons prepared with a linear velocity of 35 m/s were investigated by DSC. Figure 6 shows the DSC curves of the ribbons at a heating rate of 0.67 K/s under a highly purified argon atmosphere. Two exothermic peaks existed in each DSC curve except for the Fe_84_Si_1_B_15_ amorphous alloy. The exothermic peak of the Fe_84_Si_1_B_15_ amorphous alloy was asymmetric, which could indicate that there was no single crystallization process. We speculated that the precipitation phases of the Si-added alloys were more complicated. In the DSC curves, no obvious glass transition temperature could be detected for all alloys. The crystallization temperatures (*T*_x_) are summarized in Table 6. It could be seen that in the Fe_80+x_Si_5−x_B_15_ (*x* = 0–4) alloys, the *T*_x_ values decreased as *x* increased.

**Figure 7:**
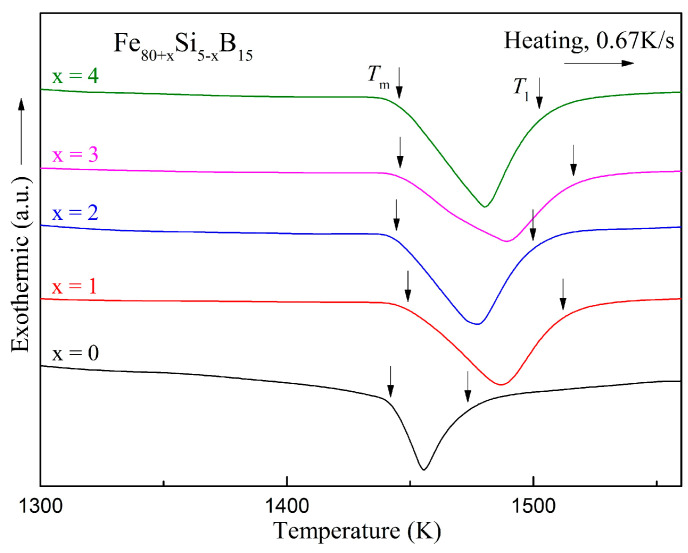
DSC curves of the melting processes for the Fe_80+x_Si_5−x_B_15_ (*x* = 0–4) alloys.

**Figure 8:**
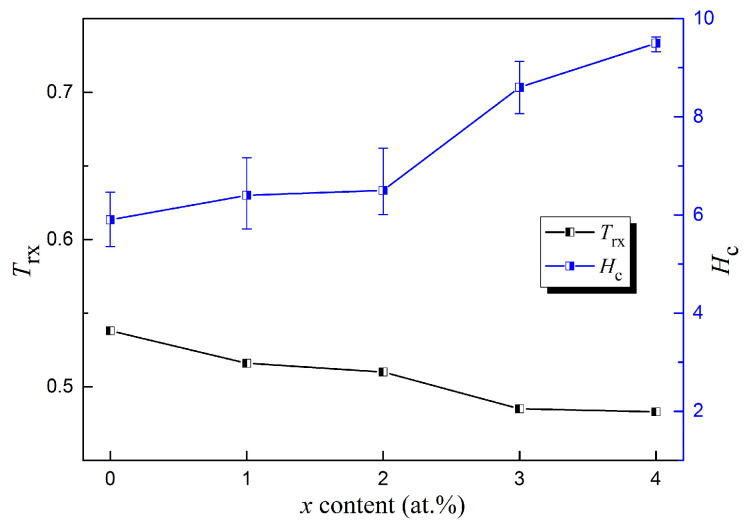
Changes in *T*_rx_ and *H*_c_ of the Fe_80+x_Si_5−x_B_15_ (*x* = 0–4) amorphous alloys.

The DSC curves of the melting processes for the Fe_80+x_Si_5−x_B_15_ (*x* = 0–4) alloys are presented in Figure 7. The onset and end temperatures of the melting endothermic events denoted by *T*_m_ and *T*_l_ are summarized in Table 6. Many parameters can be used to estimate the GFA such as the reduced glass transition temperature, *T*_rg_ = *T*_g_*/T*_l_ [42], and *γ* = [*T*_x_*/*(*T*_l_ + *T*_g_)] [43,44]. For amorphous alloys without an obvious glass transition temperature in the DSC determination, *T*_rg_ is often replaced by *T*_rx_ = *T*_x_*/T*_l_ for characterizing the GFA [8], and *H*_c_ is also an alternative because its decrease is usually accompanied by an increase in the GFA [45]. The changes of *T*_rx_ and *H*_c_ with the value of *x* for the Fe_80+x_Si_5__−__x_B_15_ (*x* = 0–4) alloys are illustrated in Figure 8 and the two parameters are added to Table 6. For *H*_c_, the values for *x* = 0–2 were close and much lower than the values for *x* = 3 and 4. For *T*_rx_, amongst the five components, *x* = 2 at.% also showed a relatively high value although it was not the highest one. From these two parameters, we could conclude that the Fe_82_Si_3_B_15_ alloy had a relatively good thermal stability and GFA.

**Figure 9:**
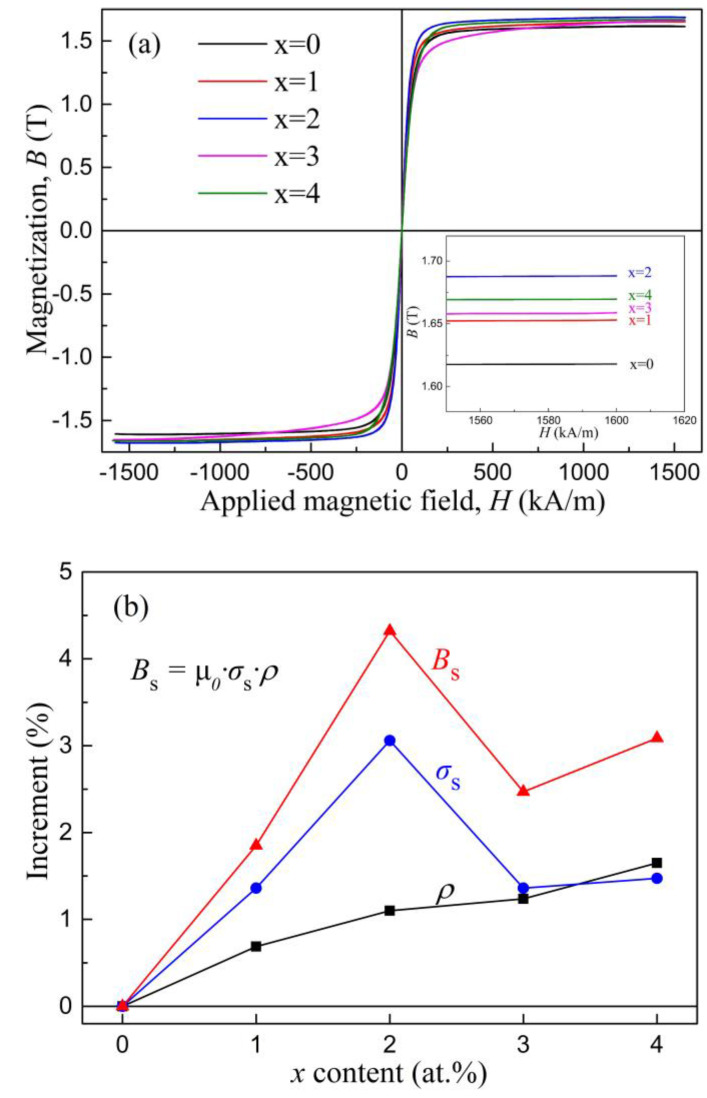
(**a**) Hysteresis loops of the Fe_80+x_Si_5−x_B_15_ (*x* = 0–4) amorphous ribbons. The insert shows the enlarged partial hysteresis curves of the samples. (**b**) The *ρ*, *σ*_s_, and *B*_s_ values as functions of Fe content.

The magnetic properties of these glassy alloys were further investigated. The hysteresis loops of the as-quenched ribbons are presented in Figure 9a. Three individual amorphous alloy ribbons of each composition, which were prepared at a linear velocity of 35 m/s and polished, were selected for the VSM test to improve the reliability of the data. All samples exhibited the typical loops of soft magnetic amorphous alloys. The *B*_s_ values were derived from the density *ρ* (measured by the Archimedean method), the permeability of the vacuum (*μ*_0_ = 4π × 10^−7^ N/A^2^), and the saturated mass magnetization *σ*_s_ according to the expression *B*_s_ = *μ*_0_·*σ*_s_·*ρ*. The results are illustrated in Figure 9b and also tabulated in Table 6. It could be seen that, with an increase of *x*, the *B*_s_ values of the amorphous ribbons first increased and then decreased. The maximum *B*_s_ value of 1.69 T was reached at *x* = 2 at.%. For the *B*_s_ of 1.69 T, the content of Si was 3 at.%, the *σ*_s_ value was 182.5 Am^2^/kg, and *ρ* was 7.36 × 10^3^ kg/m^3^. We noted that for the same sample (3 at.% Si) in the previous literature [46,47], *σ*_s_ was reported to be 177 Am^2^/kg, with *ρ* being 7.41 × 10^3^ kg/m^3^ and, accordingly, the *B*_s_ value was calculated to be 1.65 T, which was slightly lower than our result (1.69 T). Considering the possible difference in the experiments, including the ribbon quality and the testing equipment, such a slight variation was considered to be reasonable. Overall, our results indicated that when the Fe content was less than 82 at.% in the Fe-Si-B amorphous alloys, the *B*_s_ value increased with the increase in Fe content and this trend was reversed with a higher Fe content. This observation was in agreement with a previous report [8].

Combining the results of the XRD, DSC, and VSM analyses, a conclusion could be made that, amongst the Fe_80+x_Si_5−x_B_15_ (*x* = 0–4) amorphous alloys, the Fe_82_Si_3_B_15_ amorphous alloy had the highest saturation magnetization and a relatively good GFA. Considering the balance between the soft magnetic properties and the GFA, Fe_82_Si_3_B_15_ was determined to be the optimum composition. Such an experimental observation confirmed our prediction based on the simulation results.

## 4. Conclusions

In this work, the atomic structure, GFA, thermal stability, and soft magnetic properties of Fe_80+x_Si_5−x_B_15_ (*x* = 0–4) amorphous alloys were studied by a combination of AIMD simulations and experiments. The AIMD simulation results of the PDF, VP, CN, and CSRO showed that the interactions between the Fe and metalloid (M = Si, B) atoms were strong and a proper Si addition enhanced the GFA, thermal stability, and magnetic performance of the alloys. Compared with the other alloys, the Fe_82_Si_3_B_15_ amorphous alloy had relatively stable atomic clusters and featured a strong solute–solute avoidance effect, thus featuring a good thermal stability, an enhanced GFA, and good magnetic properties. To confirm the simulation results, the Fe_80+x_Si_5−x_B_15_ (*x* = 0–4) amorphous ribbons were prepared by a single roller melt-spinning method. The Fe_82_Si_3_B_15_ amorphous alloy showed a relatively good thermal stability and GFA. More importantly, it exhibited a saturated magnetization as high as 1.69 T and a low coercivity of 6.5 A/m. The investigations in this work on Fe-Si-B amorphous alloys provide a theoretical reference for further industrialization and commercialization.

## Figures and Tables

**Table 1:** Positions of the first peaks for *g*_Fe-Fe_(r) at 300 K.

Alloys	Fe_80_Si_5_B_15_	Fe_81_Si_4_B_15_	Fe_82_Si_3_B_15_	Fe_83_Si_2_B_15_	Fe_84_Si_1_B_15_
r_Fe-Fe_	2.45	2.43	2.45	2.42	2.43

**Table 2:** Fe-centered total and partial CNs in the Fe_80+x_Si_5−x_B_15_ (*x* = 0–4) alloys.

Alloys	Fe-Fe	Fe-Si	Fe-B	Total CN
Fe_80_Si_5_B_15_	12.10	0.81	1.68	14.59
Fe_81_Si_4_B_15_	12.32	0.65	1.70	14.67
Fe_82_Si_3_B_15_	12.45	0.45	1.80	14.70
Fe_83_Si_2_B_15_	12.40	0.32	1.82	14.55
Fe_84_Si_1_B_15_	12.80	0.14	1.70	14.64

**Table 3:** Si-centered total and partial CNs in the Fe_80+x_Si_5−x_B_15_ (*x* = 0–4) alloys.

Alloys	Si-Fe	Si-Si	Si-B	Total CN
Fe_80_Si_5_B_15_	12.93	0	0.76	13.69
Fe_81_Si_4_B_15_	13.09	0.04	0.27	13.40
Fe_82_Si_3_B_15_	12.26	0	0.18	12.44
Fe_83_Si_2_B_15_	13.46	0	0.29	13.75
Fe_84_Si_1_B_15_	12.02	0	0.47	12.49

**Table 4:** B-centered total and partial CNs in the Fe_80+x_Si_5−x_B_15_ (*x* = 0–4) alloys.

Alloys	B-Fe	B-Si	B-B	Total CN
Fe_80_Si_5_B_15_	8.97	0.25	0.44	9.66
Fe_81_Si_4_B_15_	9.17	0.07	0.40	9.64
Fe_82_Si_3_B_15_	9.74	0.03	0	9.77
Fe_83_Si_2_B_15_	10.08	0.04	0.37	10.49
Fe_84_Si_1_B_15_	9.50	0.03	0.27	9.80

**Table 5:** Type and fraction of atom-centered CSROs in the Fe_80+x_Si_5−x_B_15_ (*x* = 0–4) alloys.

Alloys	CSRO Type	Fe	Si	B
Fe_80_Si_5_B_15_	P-type	2.5%	34.6%	39.2%
	S-type	100.0%	0.0%	35.5%
Fe_81_Si_4_B_15_	P-type	5.8%	72.3%	59.8%
	S-type	100.0%	4.4%	40.0%
Fe_82_Si_3_B_15_	P-type	4.9%	82.4%	95.6%
	S-type	100.0%	0.0%	0.6%
Fe_83_Si_2_B_15_	P-type	5.3%	71.2%	60.5%
	S-type	100.0%	0.0%	35.6%
Fe_84_Si_1_B_15_	P-type	8.6%	52.7%	70.1%
	S-type	100.0%	0.0%	26.7%

**Table 6:** Thermal properties (*T*_x_, *T*_m_, and *T*_l_) and magnetic properties (*B*_s_ and *H*_c_) of the Fe_80+x_Si_5__−x_B_15_ (*x* = 0–4) amorphous alloys.

Compositions	Thermal Properties			Magnetic Properties		
	*T*_x_ (K)	*T*_m_ (K)	*T*_l_ (K)	*T*_rx_	*B*_s_ (T)	*H*_c_ (A/m)
Fe_80_Si_5_B_15_	793	1441	1473	0.538	1.62	5.9
Fe_81_Si_4_B_15_	781	1449	1514	0.516	1.65	6.4
Fe_82_Si_3_B_15_	765	1444	1500	0.510	1.69	6.5
Fe_83_Si_2_B_15_	735	1443	1517	0.485	1.66	8.6
Fe_84_Si_1_B_15_	728	1445	1505	0.484	1.67	9.5

## Data Availability

Data are contained within the article.
